# Development of a gene silencing DNA vector derived from a broad host range geminivirus

**DOI:** 10.1186/1746-4811-5-9

**Published:** 2009-07-02

**Authors:** Edward M Golenberg, D Noah Sather, Leandria C Hancock, Kenneth J Buckley, Natalie M Villafranco, David M Bisaro

**Affiliations:** 1 Department of Biological Sciences, Wayne State University, Detroit, MI 48202, USA; 2 Seattle Biomedical Research Institute, 307 Westlake Ave. N., Seattle, WA 98109, USA; 3 Department of Pathology and Laboratory Medicine, University of Kansas Medical Center, 3901 Rainbow Boulevard, MS1053, G017 Lied Building, Kansas City, KS 66160, USA; 4 Department of Molecular Genetics and Plant Biotechnology Center, The Ohio State University, Columbus, OH 43210, USA

## Abstract

**Background:**

Gene silencing is proving to be a powerful tool for genetic, developmental, and physiological analyses. The use of viral induced gene silencing (VIGS) offers advantages to transgenic approaches as it can be potentially applied to non-model systems for which transgenic techniques are not readily available. However, many VIGS vectors are derived from Gemini viruses that have limited host ranges. We present a new, unipartite vector that is derived from a curtovirus that has a broad host range and will be amenable to use in many non-model systems.

**Results:**

The construction of a gene silencing vector derived from the geminivirus *Beet curly top virus *(BCTV), named pWSRi, is reported. Two versions of the vector have been developed to allow application by biolistic techniques or by agro-infiltration. We demonstrate its ability to silence nuclear genes including ribulose bisphosphate carboxylase small subunit (*rbcS*), *transketolase*, the sulfur allele of magnesium chelatase (*ChlI*), and two homeotic transcription factors in spinach or tomato by generating gene-specific knock-down phenotypes. Onset of phenotypes occurred 3 to 12 weeks post-inoculation, depending on the target gene, in organs that developed after the application. The vector lacks movement genes and we found no evidence for significant spread from the site of inoculation. However, viral amplification in inoculated tissue was detected and is necessary for systemic silencing, suggesting that signals generated from active viral replicons are efficiently transported within the plant.

**Conclusion:**

The unique properties of the pWSRi vector, the ability to silence genes in meristem tissue, the separation of virus and silencing phenotypes, and the broad natural host range of BCTV, suggest that it will have wide utility.

## Introduction

Post-transcriptional gene silencing (PTGS), also known as RNA interference (RNAi) or RNA silencing, was initially described as a unique artifact of transgenic expression in petunias [1,2], but has since been established as a widespread phenomenon in many organisms [3]. The RNA silencing machinery is triggered by dsRNA molecules that are digested into small 21–24 nucleotide small interfering RNA (siRNA) fragments by DICER-like ribonucleases. These fragments are further processed and incorporated into the RNA induced silencing complex (RISC) which targets homologous mRNAs for degradation [4,5]. An interesting phenomenon particularly prominent in plants is systemic spread of RNA silencing. It is in part due to this property that RNA silencing acts as an effective natural defense against plant RNA and DNA viruses.

Experimentally, RNA silencing has been utilized to produce phenocopies of knockout strains in various organisms. In *Caenorhabditis elegans*, exposure to solutions of dsRNA is sufficient to induce silencing of the homologous gene [6]. Alternatively, injection of dsRNA directly into specimens or into fecund mothers of intended specimens (pRNAi – parental RNAi) can elicit the gene silencing response. In plants, gene silencing can be accomplished transiently by particle bombardment or agroinfiltration of hairpin (hp) RNA molecules or dsRNA, or of constructs expressing hpRNA. However, while these methods can result in systemic silencing of transgenes, only limited spread of the silencing signal is typically observed when endogenous genes are targeted. This limitation can be overcome by creating stable transgenes that express hpRNAs, but this approach can be time consuming and is not available in all species. Alternatively, vectors based on plant viruses that are themselves capable of systemic spread have been used to achieve systemic silencing of endogenous genes (virus-induced gene silencing; VIGS). Usually the viral vectors are designed to over-express a fragment of the target gene rather than a hpRNA. VIGS offers several advantages, including relative ease of use in intact model plants as well as in species that are not amenable to transgenic approaches [7-11]. Disadvantages can include incomplete and non-uniform silencing, virus host range restrictions, and complications in experimental interpretation resulting from virus replication and disease symptoms. An ideal virus vector would have an extremely broad host range and elicit a potent systemic silencing signal following replication in a limited number of cells.

Some of the most flexible VIGS systems have been developed from the geminiviruses *Tobacco curly shoot virus *(TbCSV), *Tomato yellow leaf curl China virus *(TYLCCNV) [12,13], *Tomato golden mosaic virus *(TGMV) [14,15], and *Cabbage leaf curl virus *(CaLCuV) [16]. All geminiviruses encapsidate small, circular ssDNA genomes that replicate in the host cell nucleus through dsDNA intermediates. TbCSV, TYLCCNV, TGMV, and CaLCuV are members of the genus *Begomovirus*. TbCSV and TYLCCNV are monopartite virus, while TGMV and CaLCuV have genomes consisting of two components, DNA A and DNA B. Well studied vectors based on TGMV and CaLCuV have been shown to replicate and spread systemically in inoculated plants and to support the effective silencing of endogenous genes. In addition, they provide several advantages over RNA virus vectors. Vectors based on RNA viruses sometimes generate only transient and somewhat limited silencing of endogenous genes, most likely because the virus vector is also targeted and limited by the RNA silencing system. In contrast, geminivirus vectors are not cleared by RNA silencing (although their mRNAs are targeted) and can cause efficient and durable silencing of endogenous genes even in tissues that do not support virus replication, including the meristem [14-16]. Thus, geminiviruses appear to stimulate the production of a strong systemic silencing signal. In addition, the begomoviruses collectively have a very broad host range that includes many important crop species. Unfortunately, however, the host range of individual begomoviruses is quite narrow. For example, CaLCuV is known to infect cabbage, *Arabidopsis thaliana*, and *Nicotiana benthamiana *while TGMV is limited to a few tomato and tobacco species, and primarily to *N. benthamiana *[17,18]. Other disadvantages of these begomovirus vector systems are the disease symptoms they can elicit in inoculated plants, and that both A and B genome plasmids must be co-inoculated to induce infection and silencing. Therefore cultures of helper plasmids must be maintained.

We report the construction of a gene silencing vector, pWSRi, derived from *Beet curly top virus *(BCTV), a geminivirus belonging to the genus *Curtovirus*. Curtoviruses have monopartite genomes and individual members typically have very broad host ranges. BCTV can infect the model hosts *Arabidopsis *and *N. benthamiana *and is reported to have a natural host range encompassing plants belonging to at least 41 families, including the Chenopodiaceae, Solanaceae, Brassicaceae, Asteraceae, Caryophyllaceae, Ranunculaceae, Fabaceae, Cucubitaceae, and Malvaceae (Table 1) [19,20]. Thus, the BCTV-derived vector should be useful for testing gene function over a large number and taxonomic array of dicotyledonous plants. In addition, the vector has been designed to disable spread of the virus in the plant while maintaining its ability to replicate within host cells, effectively separating gene knock-down and virus phenotypes. Lastly, the vector does not require a helper plasmid, which simplifies application. Here we demonstrate the use of pWSRito silence endogenous, nuclear genes encoding the small subunit of ribulose bisphosphate carboxylase (*rbc*S), *transketolase*, and the homeotic transcription factors *pistillata *(*SpPI*) and *apetela3 *(*SpAP3*) in cultivated spinach following biolistic delivery. We also demonstrate silencing of the sulfur allele of magnesium cheletase (*su*) in tomato following agroinfiltration of pWSRi.

**Table 1:** Subclass and 41 families of plants susceptible to *Beet curly top virus*

Asteridae	Acanthaceae
	Apocynaceae
	Asteraceae
	Boraginaceae
	Campanulaceae
	Convolvulaceae
	Dipsacaceae
	Hydrophyllaceae
	Lamiaceae
	Plantaginaceae
	Polemoniaceae
	Scrophulariaceae
	Solanaceae
	Valerianaceae
Caryophyllidae	Amaranthaceae
	Caryophyllaceae
	Chenopodiaceae
	Nyctaginaceae
	Plumbaginaceae
	Polygonaceae
	Portulacaceae
Dilleniidae	Brassicaceae
	Capparaceae
	Cucurbitaceae
	Malvaceae
	Primulaceae
	Resedaceae
	Violaceae
Hamamelidae	Cannabaceae
	Urticaceae
Magnoliidae	Papaveraceae
	Ranunculaceae
Rosidae	Apiaceae
	Euphorbiaceae
	Fabaceae
	Geraniaceae
	Linaceae
	Onagraceae
	Oxalidaceae
	Sapindaceae
	Tropaeolaceae

## Materials and methods

### Construction of BCTV VIGS vectors

**Figure 1:**
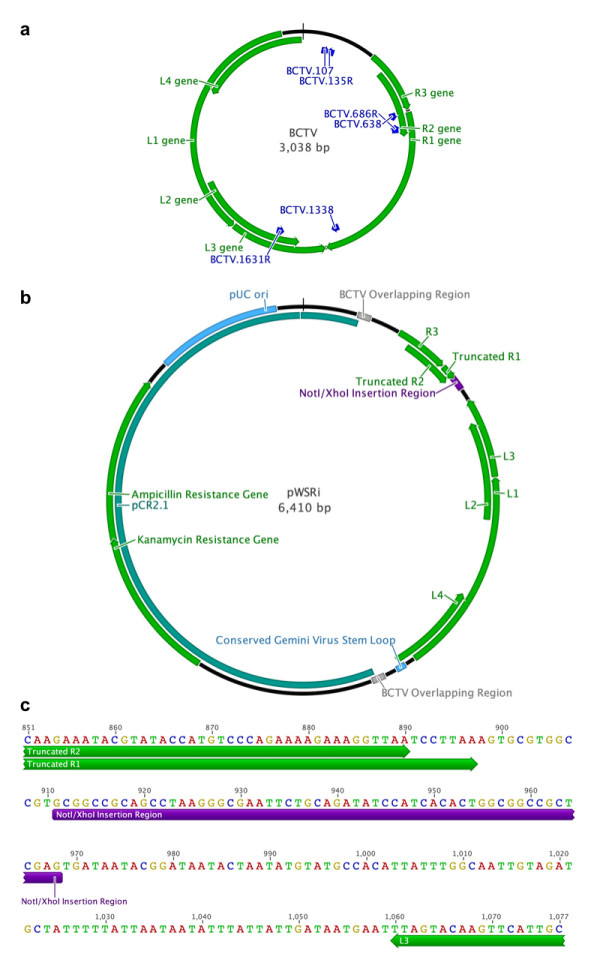
**Map of the *pWSRi *plasmid and its construction**. (a) The circular map of the BCTV genome displays the positions of the primers (blue arrows) used to construct *pWSRi *relative to the positions of the viral genes (green arrows). (b) The circular BCTV genome was inserted into a pCR2.1 plasmid vector (indicated in blue). In plant cells, the viral vector is released by recombination within the overlap (repeated) regions of the viral genome marked in red. The full-length BCTV *L1*, *L2*, *L3*, *L4*, and *R3 *are indicated with green arrows. The positions of the truncated *R1 *and *R2 *genes, interrupted by the NotI-XbaI linker for insertion of targeting sequences (in purple), are also indicated in green. Direction of the arrows indicates direction of gene transcription. (c) The nucleotide sequence near the targeting sequence insertion site is shown. The truncated R1 and R2 reading frames are denoted by arrows underneath the sequences; 14 nt were deleted from the 3' end of R2 and 626 nt were deleted from within R1. The remainder of the R1 transcription unit continues downstream of the XhoI site. The L3 reading frame, which is not truncated, is similarly marked with an arrow in the direction of the transcription. The linker containing NotI/XhoI cloning sites is shown in purple.

The pCT-2 vector was used as a source of the BCTV (Logan strain) genome [21]. Construction of the pWSRi vector was done in two steps. A fragment of the BCTV genome from position 107 to 711 (sequences and positions are based on GenBank sequence accession AF379637 and are shown in Figure 1a) was isolated by PCR. This fragment contains the complete R3 gene from position 344 to 610, most of the R2 gene, which normally is from 420 to 728, and the beginning of the R1 gene which starts at position 640. The primers used in the reaction were BCTV.107 5' AGG CTG AGC TCT TTC AGA TAA GAT TTG TTG ACT 3' and BCTV.686R 5' AGG CTG CGG CCG CAC GGC CAC GCA CTT TAA GGA TTA ACC T 3'. Both primers were designed to have a 5–6 base 5' lead sequence followed by a *SacI *restriction site in BCTV.107 and a *NotI *site in BCTV.686R. BCTV.686R was also designed to deviate from the AF379637 sequence at position 690 (G to T) and at position 697 (C to T). The change at position 690 introduces a stop codon in the R2 gene, which truncates the protein sequence by 12 amino acids. The change at position 697 introduces a stop codon in the R1 gene resulting in a short open reading frame that encodes a polypeptide of only 19 amino acids. This fragment was cloned into the pCR 2.1 vector (Invitrogen, Carlsbad, CA) using TOPO TA cloning. The second fragment of the BCTV genome that was isolated by PCR included positions 1338 to position 155. The entire genome is circular and is 3038 bp in length, therefore this fragment was 1856 bp in length. This fragment included the 3' end of the R1 gene, which ends at position 1404, the L3 gene from 1839 to 1429, the L2 gene from 2068 to 1547, the L1 gene from 2936 to 1860, and the L4 gene from 2779 to 2522. The fragment was also designed to have a 49 bp overlapping fragment with the first fragment. The primers used to amplify the reaction were BCTV.1338 5' AGG CTG CTC GAG TGA TAA TAC GGA TAA TAC TAA 3' and BCTV.135R 5' AGG CTT CTA GAA ATA AAG TTG TCC CCT TCT AT 3' (Figure 1a). As with the previous primers, these primers had a 5' leader sequence followed by an *XhoI *and an *XbaI *restriction sites, respectively. This fragment was cloned into the pCR4 vector (Invitrogen, Carlsbad, CA) following the manufacturer's protocol. All clones were sequenced to verify the correct insertions.

The two constructs were digested with *XhoI *and *XbaI *restriction enzymes following the manufacturer's protocol (Promega, Madison, WI). The digested fragments were size separated on a 1.5% agarose gel. The BCTV fragment 1338-155 and the pCR2.1:BCTV107-711 fragment were cut from the gel and extracted using a Qiagen Gel Extraction Kit (Qiagen, Valencia, CA). The fragments were ligated overnight using T4 DNA ligase at 16°C. The resultant construct, called pWSRi, was tested by a restriction digest using *SacI *and *XbaI*. Positive clones were then verified by sequencing.

A second plasmid was constructed by cloning a fragment from position 638 to position 1646. The primers used for amplification were BCTV. 638 5' AAG TGT TCG TTG TAT GAG G 3' and BCTV.1631R 5' AGT GCT GGA TAG ATT TGA C 3' (Figure 1a). The BCTV.1631R sequence was derived from the sequence found in the more aggressive accession U02311 [22], but was sufficiently similar to the Logan strain to allow amplification. This region includes the 3' end of the R2 gene, the entire R1 gene, and the 3' ends of the L1 and L2. The fragment was cloned into the pCR2.1 vector using TOPO TA cloning. The insert region was verified by sequencing.

The pWSRiL1^- ^plasmid was constructed to have a G to T transversion in the start codon of the BCTV *L1 *gene. Two sets of primers, pWSRi.2236 (5' ATT GTG TCT CCG TTC TTG TC 3') and pWSRi.2556Rmut (5' GGA GTC CAA TT*C CTC CTA CT 3') and pWSRi.2556mut (5' AGT AGG AGG A*AT TGG ACT CC 3') and pWSRi.2836R (5' ACT CAC TAT AGG GCG AAT TG 3') were designed to amplify overlapping fragments of the pWSRi vector such that the overlapping ends would include the mutated *L1 *start codon (* base). The two fragments were linked together to form a 619 bp fragment by employing both as template in a PCR reaction using the outside primers. *SalI *and *XbaI *recognition sites are located internal to both primers in the fragment and are found at positions 2253 and 2825 in pWSRi. The mutated PCR fragment was then switched with the native fragment in pWSRi, by double digesting both with *SalI *and *XbaI*, gel purifying the cut plasmid and fragments, and ligating with T4 DNA ligase. The modified pWSRi plasmid was verified for the mutation by sequencing and was designated pWSRiL1^-^.

The pWSRi vector was also transferred to a binary Ti plasmid to allow agroinfiltration. To do this, the viral sequence was tagged with a chloramphenicol acetyl transferase (CAT) gene by inserting the CAT sequence into the *NotI*-*XhoI *insertion sites in the vector. The presence of CAT facilitated transfer of the BCTV sequence because it allowed selection of positive clones. CAT (863 bp) flanked by *NotI *and *PacI *restriction sites at the 5' end, and by *AscI *and *XhoI *sites at the 3' end, was obtained by PCR from pACYC184 using the primers 5' GCG CGG CCG CTT AAT TAA CTT TTG GCG AAA ATG AG 3' and 5' GCG CTC GAG GGC GCG CCG CCA TTC ATC CGC TTA TTA TC 3'. The BCTV sequence containing CAT was excised using *HindIII *and *XbaI *and inserted into a similarly cleaved pBI121 derivative plasmid lacking the 35S-GUS-3' sequence. The resulting vector was designated pWSRiA:CAT. Other targeting sequences can be easily inserted into this base vector simply by replacing CAT and screening for chloramphenicol-sensitive colonies.

### Isolation and insertion of targeting sequences

Segments of three spinach nuclear genes, *rbcS*, *transketolase*, and *SpPI*, were isolated by PCR and cloned into pCR4 using TOPO TA cloning. The *rbcS *primers were designed from the Genbank accession L76557[23] and were designated SprbcS.114XhoI 5' AGC TCG AGC ACC AAG AAG AAC GAT GAC A 3' and SprbcS.505RPstI 5' agc tgc agg caa tga aac tga cac a 3'. The *transketolase *primers were designed from the Genbank accession L76554[24] and were designated SpTrnktls.711XhoI 5' CTC GAG GGA AGG GAT TGC TCA GGA AG 3' and SpTrnsktls. 937RNotI 5' gcg gcc gca aag tgg gtt tgt ctg tga c 3'. The *rbcS *and *transketolase *PCR fragments were cloned into the pGEM T-easy vector using T4 DNA ligase following manufacturer's protocols (Promega, Madison, WI). The *SpPI *fragment was digested from a plasmid designated *pSpPI, c *that included the 3' end of the cDNA from position 526 to the poly-A tail (255 bp) and cloned into pBluescript SK (Stratagene, La Jolla, CA) (Genbank accession AY604515) [25]. The inserts were then subcloned into pWSRi by double digestion with *NotI *and *XhoI*. The digested fragments were separated on a 1.5% agarose TBE gel, the appropriate DNA bands were excised, and the DNA fragments were isolated using the Qiagen Gel Extraction Kit (Qiagen, Valencia, CA). The gene fragments were individually cloned into the *NotI/XhoI *digested pWSRi vector using T4 DNA ligase overnight at 16°C. The orientation of the *NotI/XhoI *sites resulted in all three inserts being in the antisense orientation relative to *R1 *gene. The ligations were transformed into JM109 chemically competent cells. Transformants were screened and positive clones were verified by DNA sequencing.

pWSRi:SpAP3 was constructed by subcloning a 3' region from position 432 to 834 (403 bp) of the gene previously cloned and used for gene specific *in situ *hybridization studies [25]. As in the previous construct, the appropriate fragment was size separated by electrophoresis and extracted from the gel. Ligation into the *NotI/XhoI *sites resulted in *SpAp3 *being in an antisense orientation relative to *R1*.

To create pWSRiA:su, a 999 bp fragment (nucleotides 250–1249) of the *Arabidopsis thaliana ChlI *gene was obtained from pTV:09 by PCR using the primers 5' GCG TTA ATT AAA GGC GAG ACC GGT TTA TCC 3' and 5' GCG GGC GCG CCC GGA AAC TAG AAC TCC TGA A 3'. The pTV:09 vector was kindly provided by David Baulcombe. The use of these primers placed a *PacI *site at the 5' end, and an *AscI *site at the 3' end, of the *ChlI *sequence. The cleaved *ChlI *sequence was then used to replace the CAT gene in similarly cleaved pWSRiA:CAT. The resulting construct, which contains *ChlI *sequence in the sense orientation relative to the viral *R1 *gene, was designated pWSRiA:su.

### Biolistic bombardment of plants

Spinach plants (*Spinacea oleracea *cv. America) were grown in a growth chamber at 22–23° C under a 16 hour light cycle. A particle inflow gun (gene gun) was constructed following the design of Vain et al. [26]. Plasmid preps were made of the pWSRi constructs using the Wizard SV Miniprep Kit (Promega, Madison, WI). One hundred fifty milligrams of tungsten particles were sterilized by incubating in 100% ethanol for thirty minutes. The particles were then washed three times in sterile water and resuspended in a final volume of 1.5 ml sterile water. The plasmid DNA was precipitated onto the tungsten particles by combining 200 μl of the tungsten/water slurry, 25 μl plasmid extractions, 200 μl 2.5 M CaCl_2_, and 8 μl 860 mM spermidine, and incubating at 4°C for 1 hour. Fresh spermidine solution appears to be critical. The slurry was then centrifuged and 200 μl of the liquid was removed. The remaining slurry was vortexed and 20 μl were applied to the screen of the luer lock adapter of the particle gun. Seedlings having two cotyledons and two true leaves were placed in the vacuum chamber approximately 15–20 cm from the adapter. A vacuum was drawn and the particles were expelled onto the plant by helium at 60 psi pressure. Plants were returned to the growth chamber and covered with plastic wrap for two days to prevent desiccation during recovery. Initial treatments were designed to detect silencing of *rbc*S and *transketolase*. Treatment conditions are listed in Table 2 and include four negative controls and four test conditions.

**Table 2:** Silencing of *rbcS *and *transketolase*

Treatment^a^	Plants silenced after treatment
a. Initial Trials	
1. no DNA (mock)	0 of 6 plants
2. *pWSRi*	0 of 6 plants
3. *pBCTV.R1*	0 of 6 plants
4. *pWSRi *+ *pBCTV.R1*	0 of 6 plants
5. *pWSRi:rbcS*.	6 of 6 plants
6. *pWSRi:rbcS *+ *pBCTV.R1*	6 of 6 plants
7. *pWSRi:transketolase*	6 of 6 plants
8. *pWSRi:transketolase *+ *pBCTV.R1*	6 of 6 plants
	
b. Secondary Trials	
*pWSRi:rbcS*.	12 strong to moderate
	5 medium to light
	1 no response
*pWSRi:transketolase*	12 strong to moderate
	3 no response
	3 dead

^a^Tungsten particles were precipitated with the indicated DNAs and used to biolistically inoculate spinach plants. Plants were scored for leaf bleaching indicative of silencing beginning 6 weeks post-inoculation.

Plants were also treated by an alternative biolistic protocol using the Helios Gene Gun (Bio-Rad, Hercules, CA). In this protocol, 50 μl of plasmids were mixed with 20 mg of sterilized tungsten powder. The slurry was mixed well and spread on a microscope slide to allow the water to evaporate. The tungsten was then funneled into PVP-coated tubing for bullet preparation. Bullets were fired from the Helios Gene Gun at 90 psi of helium at a distance of 1–3 cm.

### Agroinfiltration of tomato

Tomato plants (*Solanum lycopersicum *cv. Rutgers) were grown in a growth chamber at 24°C under a 14 hour light cycle. Agroinfiltration was carried out as previously described (Wang et al., 2005) Briefly, the pWSRiA:CAT and pWSRiA:ChlI vectors were transformed into *Agrobacterium tumefaciens *strain C51c. Cultures were grown overnight at 30° in medium containing 20 μM acetosyringone, and resuspended to OD_600 _= 1 in 10 mM MES buffer containing 10 mM MgCl_2 _and 20 μM acetosyringone. Cells were allowed to stand in the buffer for 3 hours prior to infiltration onto the underside of cotyledons and leaves of tomato seedlings (two to four leaf stage) using a 1 ml syringe lacking a needle.

### Primer design for PCR assay of viral infection and vector transport

Total nucleic acids were extracted from whole spinach leaves that demonstrated bleaching following earlier pWSRi:SprbcS treatment using a standard CTAB DNA extraction protocol. SprbcS.114XhoI and SprbcS.505RPstI primers were used as positive controls on both leaf and plasmid DNAs. SprbcS.505R and pWSRi.1338R (5' TTA GTA TTA TCC GTA TTA TCA CTC GAG CAG CCT 3') primers were used to assay the presence of pWSRi:rbcS DNA. pWSRi.1338R directly flanks the XhoI insertion site in the pWSRiplasmid and so can be used with an insertion sequence specific primer to verify the presence of the cloned insertion sequence within pWSRi. To detect evidence of recombination and transposition of viral sequences of the pWSRi vector constructs, we designed two new primers, BCTV. 3007 (5' CAA CTC TCA TAA GGG CCA TC 3') and BCTV.177R (5' GGG GCC CAC TAA CTT TAC TT 3'). The two primers are located in the L and R fragments of the pWSRi vector and are separated by the pCR2.1 plasmid sequences. In subsequent trials in which pWSRiL1^- ^replication was tested, we used pWSRi.2236 (5' ATT GTG TCT CCG TTC TTG TC 3') and pWSRi.2556R (5' GGA GTC CAA TAC CTC CTA CT 3'). These primers are in the L region and can amplify both pWSRi and pWSRiL1^-^.

### Quantification of mRNA knockdown by quantitative RT-PCR

Total RNA was extracted from leaves of pWSRiA:ChlI and pWSRiA:CAT treated plants four weeks after agroinfiltration using Trizol following the manufacturer's protocol. Concentration of the total RNA was estimated by spectrophotometry. Five ul of each RNA extract was mixed with 2 ul 100 uM random hexamers, 1 ul RNase Inhibitors and nuclease free water to a volume of 20 ul. The samples were heated to 70°C for five minutes and then placed on ice. A master mix consisting of 10 ul M-MLV RT 5× Buffer, 2.5 ul 10 mM dNTP, 15.5 ul water, and 2 ul M-MLV Reverse Transcriptase was added to each sample. The samples were left at room temperature for 10 minutes and then incubated at 55°C for an additional 50 minutes. One ul RNase H was then added and the samples were incubated at 37°C for 20 minutes.

The aliquots of each sample were then diluted in nuclease free water based on the original concentrations of total RNA such that the cDNAs from 2.5 ng RNA were diluted to a final volume of 50 ul. 2× master sybr green PCR mixes minus the template and primers for nine 20 ul PCR reactions were prepared for each sample. Eighteen ul (2 ul per reaction) of the diluted cDNAs were then added to the master mixes and mixed. This batch mixing assured that each control and experimental PCR reaction would have the same concentration of cDNA within each sample trial. The reaction volumes were then divided into two tubes (4 reactions each), and 8 ul of 2.5 uM forward and reverse primer pairs were added. For the experimental reactions, two primers were designed from the *Solanum lycopersicum *sequence BT012789 to produce a 394 bp product (LeChelatase.630 5'-TGG GAC AAT CGA CAT TGA GA-3' and LeChelatase.1023R 5'-TTC TTG CTC CCC CTT GTA TG-3'). Ambion QuantumRNA Universal 18S primers were mixed in a 4:6 ratio with the 18S competimers and used for the internal control reactions. The expected PCR product would be 324 bp in length. The two master mixes (Mg-Chelatase and 18S) were then aliquoted into three replicate wells each on a 96 well plate, with the three replicates staggered over the plate. Thus each cDNA sample was measured in three replicates for each of the *Mg Chelatase *(*su*) and *18S *control primer pairs. The PCR temperature settings were 94°C for 10 minutes followed by 40 cycles of 94°C 15 seconds, 60°C 15 seconds, and 72°C 45 seconds, and reactions were run and the data collected on a Stratagene Mx3000P. Mean threshold cycles for the Mg-Chelatase (*su*) and 18S were calculated for each sample. The delta CT (Threshold cycle_Chelatase _-Threshold cycle_18S_) was calculated from the means and the delta CT variances were calculated by summing the individual CT variances as there should be no covariance of the sample errors.

## Results and discussion

### Construction and description of pWSRi

A plasmid vector was constructed by cloning two fragments of BCTV into a bacterial plasmid vector (pCR 2.1; Invitrogen). The first fragment included the viral *R3 *gene and the 5' portions of the *R2 *and *R1 *genes. *R1 *(also known as *V1*) directs synthesis of the capsid protein and *R2 *(*V2*) encodes a protein required for the efficient accumulation of viral ssDNA. Both *R1 *and *R3 *(*V3*) are required for spread of the virus in the infected plant [27,28]. Nonsense mutations were introduced into the already truncated *R2 *and *R1 *genes. The *R2 *open reading frame is truncated by 12 amino acids, while the *R1 *gene encodes only 19 amino acids. These mutations are intended to prevent virion production and viral movement within the plant. The second fragment included a 3' fragment of *R1*, the intact *L1*, *L2*, *L3*, and *L4 *genes, as well as the viral origin of replication encompassing the conserved hairpin loop structure [29]. *L1 *(also known as *C1 *or *Rep*) is the only viral gene required for replication. *L3 *(*C3*) encodes the replication enhancer (REn), and *L2 *(*C2*) specifies a protein that is important for pathogenesis and has silencing suppressor activity. The *L4 *gene also specifies a pathogenicity factor [21,27,30-34]. An overlapping region between the two viral fragments was included to allow intramolecular recombination. Thus a fully replication-competent, but truncated BCTV genome (the viral vector) is released from the plasmid vector following recombination in plant cells [35]. However, the absence of R2 function minimizes the accumulation of viral ssDNA and the absence of R1 (capsid protein and movement factor) precludes virion production and virus spread.

Further modifications were designed to place the insertion site for targeting sequences and to prevent translation of spurious fusion proteins. The NotI-XhoI insertion sites for targeting sequences were introduced 14 bp downstream of the introduced *R1 *stop codon and 64 bp before the R1 natural stop codon to retain mRNA processing signals. Thus the targeting sequence should be transcribed as part of the rightward transcription unit that includes the *R1 *gene [36]. The natural stop codon of the *L3 *gene is on the opposite strand and is 92 bp from the XhoI cloning site. Because of the stop codons in the *R1, R2*, and *L3 *reading frames, no fusion proteins should be produced. However, placement of the Not1-XhoI insertion sites near the termini of two converging transcription units should favor transcription of complementary RNAs containing the targeting sequence. The completed plasmid was designated pWSRi. A map of the plasmid is shown in Figure 1b and sequences in the vicinity of the targeting sequence insertion site are given in Figure 1c.

To test whether the viral capsid protein is necessary for or can enhance silencing activity, possibly by supporting at least limited virus spread, a second plasmid was constructed by cloning a segment of BCTV from position 638 to position 1649 into pCR2.1. This putative helper plasmid was designed to express the complete R1 capsid-encoding gene and was designated pBCTV.R1.

### Silencing of endogenous nuclear genes encoding photosynthetic enzymes in spinach

**Figure 2:**
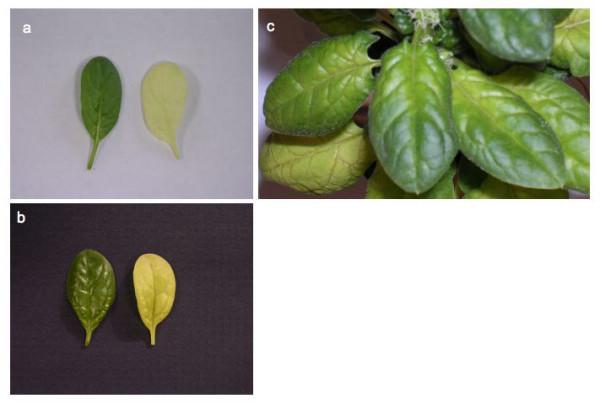
**Silencing phenotypes obtained with pWSRi:rbcS and pWSRi:trnktls display bleached leaves**. (a) Leaves from spinach plants treated with pWSRi vector (left) or pWSRi:rbcS (right). (b) Individual leaves from plants treated with pWSRi vector (left) or pWSRi:trnktls (right). The pWSRi:rbcS-treated leaf is yellow-white, whereas pWSRi:trnktls-treated leaf is yellow with residual green color often evident in the veins. (c) Photograph of pWSRi:rbcS treated-plant showing progressive bleaching of the leaves from the petiole, spreading into the blade and distally along the midrib.

We first chose to test the BCTV vector in cultivated spinach (*Spinacea oleracea*), a species for which reliable transgenic technology is not yet available. A 407 bp fragment of the spinach *rbcS *gene and a 245 bp fragment of the spinach *transketolase (trnktls) *gene were cloned into the NotI-XhoI cloning site within the truncated *R1 *gene in pWSRi. The two constructs were designated pWSRi:rbcS and pWSRi:trnktls. Both *rbc*S and *transketolase *genes have been used as silencing reporter genes in *Arabidopsis *and *Nicotiana *systems [7,37,38]. As both genes encode proteins involved in the Calvin cycle, interference with their products results in bleaching of the leaf. In initial trials, six plants were biolistically bombarded at the two leaf-two cotyledon stage with tungsten particles for each treatment listed in Table 2a. There were no observable differences among plants inoculated with empty vector (pWSRi), plants treated with tungsten particles that were not precipitated with DNA, and wild-type untreated plants. However, approximately six to eight weeks after treatment, plants receiving treatments 5, 6, 7, or 8 (pWSRi vectors with target gene inserts with and without the pBCTV.R1 helper plasmid) began to show evidence of bleaching. All plants treated with the *rbcS *containing vector developed white leaves and there was no readily apparent difference between plants treated with or without pBCTV.R1 in addition to pWSRi:rbcS (Figure 2a). The leaves on all plants receiving treatments with *transketolase*-containing vectors developed a light yellow color with leaf veins remaining green for another several days (Figure 2b). As with the *rbcS *treated plants, there was no apparent difference between treatments with or without pBCTV.R1. Thus the pWSRi:trnktls and pWSRi:rbcS constructs successfully generated similar but distinct phenotypes that mimic a photosynthetic mutant. We further concluded that our helper plasmid encoding the viral capsid protein was not necessary and did not obviously enhance vector efficacy. It should be noted that we also did not observe any obvious signs of active viral symptoms, which would be expected if an active BCTV virus were mobile, nor did we assay for capsid protein. Therefore, we cannot conclusively rule out that the pBCTV.R1 was ineffective in expressing the capsid protein.

Subsequent trials were performed without the pBCTV.R1 plasmid (Table 2b). Three sets of 6 plants were treated on different occasions with pWSRi:trnkls. In total, 12 of the 18 plants showed strong to moderate bleaching, three plants displayed no bleaching, and three plants died after treatment most likely due to the trauma of the biolistic treatment. Three additional sets of 6 plants were treated with pWSRi:rbcS. Twelve plants developed strong to medium bleaching, five plants displayed moderate bleaching, and one plant showed no response.

In treatments with pWSRi:rbcS and pWSRi:trnktl, bleaching first appeared on the newest leaves and later in older leaves until most leaves above the inoculation site were affected. In older leaves, progressive bleaching could sometimes be seen beginning at the petiole and spreading into the leaf blade and distally along the midrib, until most or all of the leaf was affected (Figure 2c). Following treatments with pWSRi, pWSRi:rbcS, and pWSRi:trnktls there was no evidence of BCTV disease symptoms which include upwardly rolled leaf margins, deformation and curling of younger leaves, vein swelling, and stunting. Wild-type BCTV is normally restricted to the phloem and, by design, the movement-defective BCTV vector is unable to spread from the site of inoculation. The absence of disease symptoms is consistent with this expectation. Thus the observation of bleaching throughout the leaf blade, including mesophyll tissues, indicated that silencing of *rbcS *and *transketolase *was due to systemic propagation of durable and/or distally amplified signals initiated from the locally confined virus vector.

### Silencing of genes expressed in the floral meristem

To confirm that silencing signals were systemically propagated from the BCTV vector, we asked whether it could be used to silence genes in meristematic tissue, from which geminiviruses are normally excluded. We first tested fragments from the spinach B class floral identity genes *PISTILLATA *(*SpPI*) and *APETALA3 *(*SpAP3*) in pWSRi [25]. According to the angiosperm ABC model, B class genes are necessary for the development of petals and stamens [39]. However, normal dioecious spinach plants have only male or female flowers. Further, in spinach flowers petals are completely absent in both sexes, and the stamen whorl is additionally absent in female flowers. Thus in spinach, *PI *and *AP3 *function is required and these genes are expressed only in male flowers [25].

**Figure 3:**
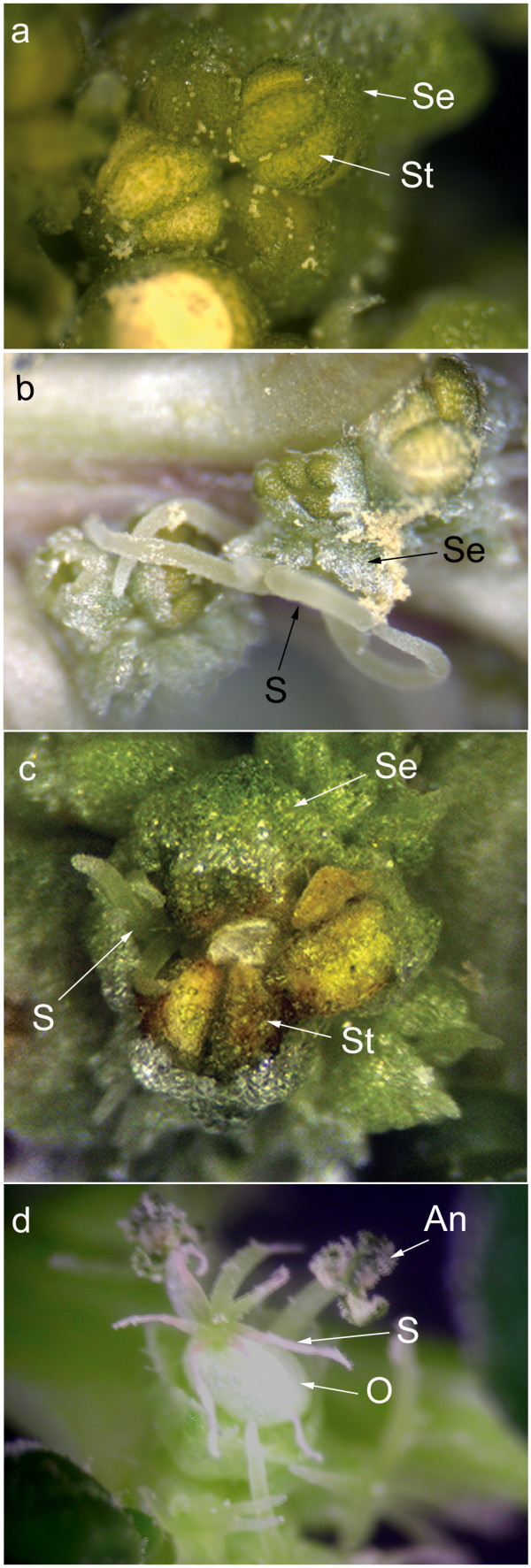
**A sample of flower morphologies found on plants bombarded with pWSRi:SpPI or pWSRi:SpAP3 display modified organ development**. a. Normal male flower within a male inflorescence from a wildtype plant. The flower is made up of four sepals that cup four stamens developing opposite to them (one pair marked with arrows). No petals or carpels are present or initiated in wild type male flowers. b. Phenotypically normal female flower among a cluster of male flowers on a plant treated with pWSRi:SpPI. The flower consists of two large sepals (arrow) that envelop the single carpel gynoecium. Stigmas (arrow) extend out from between the sepals. c. Single flower with a pistil developing across from a stamen. The flower is found in a cluster of normal male flowers on a plant treated with pWSRi:SpAP3. d. A flower with an exposed swollen ovary (no enclosing sepals) and with shriveled stigma on a pWSRi:SpPI treated plant. The size of the ovary and stigma are consistent with development post-fertilization. Two filaments with dehisced anthers from the same flower can be seen going back into the picture. St: stamen; Se: sepal; S: stigma; O: ovary, An: Anther.

Twenty-six plants at the two leaf-two cotyledon stage were bombarded with tungsten particles that had been precipitated with the pWSRi:SpPIvector. Six to eight weeks later, at the time of flowering, the sex of each plant was determined based on the inflorescence architecture. Plants bombarded with mock-precipitated tungsten particles or particles precipitated with empty pWSRi vector were used as negative controls, and in no case did these treatments result in altered flower development (not shown). However, all male plants that received pWSRi:SpPI began to develop diverse floral morphologies after eight weeks (Table 3). On these male plants, wild-type male flowers with four sepals and four stamens were found along with apparently wild-type female flowers (two sepals and one carpel/pistil), flowers with stamens and carpels in the third whorl, and flowers with four sepals and four carpels in the third whorl (Figure 3). These results are consistent with a model in which the *SpPI *gene is silenced individually in each flower at different developmental stages. In contrast, female plants had normal female flowers and did not display any apparent morphological deviations in flowers or in any other tissues. Further, in both male and female plants there was no evidence of BCTV disease symptoms in any tissues. In flowers, BCTV symptoms usually appear as severe deformation of all floral parts without homeotic transformation.

**Table 3:** Silencing with *pWSRi*:floral homeotic gene constructs

Construct	Plants Treated	Results
pWSRi:SpPI	26	12 normal females
		14 males with mixed floral organs
		
pWSRi:SpAP3	18	7 normal females
		1 normal male
		10 males with mixed floral organs

Spinach plants were biolistically inoculated with the indicated constructs. Plant sex was determined 6–8 weeks post-inoculation, and floral phenotypes visually scored for male or female characteristics.

Similar results were obtained following inoculation with pWSRi:SpAP3. Flowers exhibiting a range of phenotypes, including mixed male and female characteristics (Figure 3d), were observed only in male plants and not in female plants (Table 3). Thus the effects of inoculation with pWSRi:SpPI and pWSRi:SpAP3 were entirely consistent with the known functions of these B class genes and with their patterns of expression in spinach [25].

In summary, we observed specific and predictable phenotypic effects following inoculation of spinach plants with pWSRi constructs targeting Calvin cycle enzyme-coding genes as well as different B class floral identity genes. This allowed us to conclude that the pWSRi vector can be used to generate durable systemic silencing signals effective against endogenous genes of different types and expressed in different tissues, in absence of virus disease symptoms.

### Viral DNA replication, but not virus spread, is required for silencing

**Figure 4:**
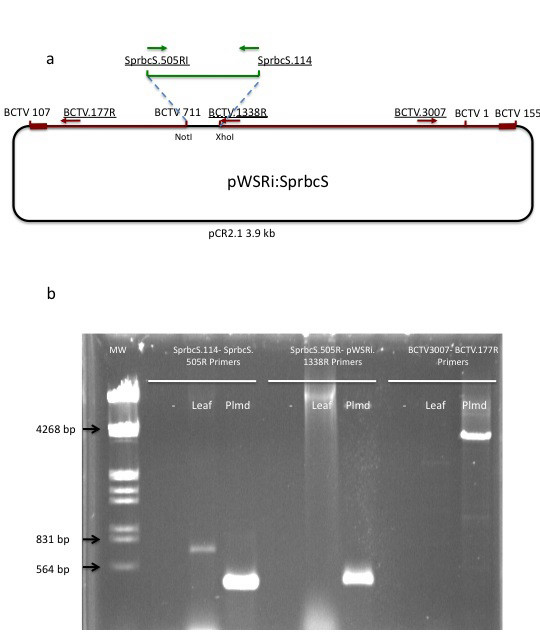
**pWSRi and BCTV genomes released from pWSRi are not detected in systemically silenced leaves distal to the inoculation site**. a. The *pWSRi:SprbcS *plasmid is presented (not to scale). The pCR2.1 sequence is indicated in black. The two BCTV regions are indicated in red, with the overlapping region (positions 107 to 155) marked with a thicker red line. The *SprbcS *insert is indicated by a green line and is found between the NotI and XhoI cut sites at positions 711 and 1338 of the BCTV sequence, respectively. Primers are underlined and their locations and direction are indicated by arrows. The SprbcS.505R and SprbcS.114 primers are indicated by green arrows and are located above the complementary positions in the *SprbcS *insert (green). The BCTV.1338R primer (red) is located next to the XhoI cloning site. The BCTV.3007 primer is located to the left of positions 3038/1 in the right BCTV insert. The primer BCTV.177R is located in the left BCTV insert, to the right of the overlap region. b. DNA was obtained from bleaching leaves of plants treated six weeks previously with pWSRi:rbcS. An agarose gel showing PCR products obtained using three different primers sets is shown. The SprbcS.114-SprbcS.505R primers were designed to detect *rbcS *sequences present in either the spinach genome or the vector. The SprbcS.505R-BCTV.1338R primer pair was specific for a pWSRi:rbcS construct. The BCTV.3007-BCTV.177R primer set was specific for released viral DNA or for amplification of the viral DNA and the plasmid backbone. Lane 1, HindIII/EcoRI digested lambda DNA size markers. Remaining lanes show PCR products obtained using SprbcS.114-SprbcS.505R primers (lanes 2–4); SprbcS.505R-BCTV.1338R primers (lanes 5–7); or BCTV.3007-BCTV.177R primers (lanes 8–10) (3 minute extension times). Lanes 2, 5, and 8 (-) are negative controls (water as template). Lanes 3, 6, 9 (Leaf) are reactions using DNA extracted from *rbc*S silenced leaves. Lanes 4, 7, and 10 (Plmd) are reactions using pWSRi:rbcS plasmid DNA.

To assess whether silencing requires the physical presence of the full pWSRi plasmid vector or the recombined and released BCTV viral vector, DNA was extracted from bleached leaves of pWSRi:rbcS treated plants which were not yet present at the time of inoculation. Positive control reactions using a primer pair (SprbcS.114, SprbcS505R, Figure 4a) designed to amplify *rbcS *confirmed the presence of this sequence in both genomic DNA from the leaf extract and in a pWSRi:rbcS plasmid preparation (Figure 4b, lanes 2–4). The amplified fragment from the leaf extract is larger than that from the pWSRi:rbcS plasmid due to the presence of an intron in the genomic sequence. The *rbcS *insert in the plasmid was derived from cDNA. Therefore, the absence of the smaller cDNA band in the leaf extract indicates that no pWSRi:rbcS or excised BCTV:rbcS traces are detectable in the effected leaf. Similarly, PCR amplification using an *rbc*S primer and a pWSRi primer that flanks the insertion site (SprbcS505R, BCTV.1338R, Figure 4a) produced a product only from the pWSRi:rbcS plasmid preparation and not in the leaf extract DNA (Figure 4b, lanes 5–7). This supports the previous conclusion that neither a complete pWSRi:rbcS plasmid nor a recombined BCTV genome with the inserted rbcS sequence are present in the bleached leaf. A pair of internal BCTV primers (BCTV.3007, BCTV.177R, Figure 4a) were designed to generate expected a 250 bp amplification product from a released viral genome or a 4 kb product from a complete pWSRi plasmid. We anticipated that the released viral genome would be rare, especially in the plasmid preps themselves. Therefore, we executed separate PCR reactions either with short extension times (45 seconds) which would favor a 250 bp product over the larger product or long extension times (3 minutes) to favor larger products. In the short extension time reactions, a light 250 bp product band was detected from the plasmid preparation, and was also observed using additional diagnostic primer pairs pWSRi.2236 and pWSRi.2556R (data not shown). The simplest interpretation is that some recombination resulting in release of the viral genome occurs in bacterial cells. No products were detected in the leaf DNA. Under longer extension times, a dominant 4 kb product was amplified from the plasmid itself but not from the leaf DNA (Figure 4b, lanes 8–10). In summary, we were not able to detect the pWSRi:rbcS or BCTV sequences in the affected leaf. This is consistent with deletion of the BCTV *R1 *gene required for both virus movement and virion formation, and confirms the separation of virus and silencing phenotypes. However, it must be recognized that although the presence of viral DNA could not be detected, it is still possible that trace amounts were present.

**Figure 5:**
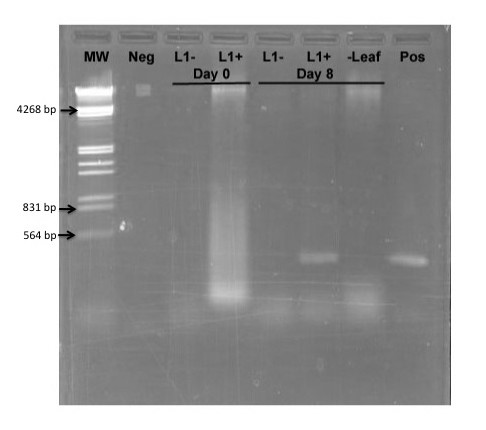
**Inactivation of the *L1 *(Rep) gene in pWSRi abolishes replication**. Individual leaves of spinach plants were inoculated with either pWSRi (L1^+^) or pWSRi L1^-^. DNA was extracted from inoculated leaves and adjacent leaves immediately after biolistic application (Day 0) or 8 days later and used as template for PCR using pWSRi.2236 and pWSRi.2556R primers designed to amplify viral DNA. L1- indicates template DNA from leaves treated with pWSRiL1^-^. L1+ indicates template DNA from leaves treated with pWSRi. The sample marked (-Leaf) indicates template DNA from an untreated leaf on the pWSRi treated plant. Neg refers to PCR reactions without template and Pos indicates PCR reactions using the pWSRi plasmid DNA as a template. MW indicates HindIII/EcoRI digested lambda DNA.

To determine whether or not replication is necessary to generate a silencing signal, we constructed a new plasmid in which the *L1 *gene, necessary for viral replication, was disabled by mutation of the start codon. This plasmid was designated pWSRiL1^-^. The pWSRi and pWSRiL1^- ^plasmids were dried onto tungsten particles and applied to individual leaves on separate plants with a BioRAD Helios Gene gun. DNA was extracted from inoculated leaves immediately following application or eight days later. Additionally, DNA was extracted from untreated leaves adjacent to those inoculated with pWSRi on the eighth day. Presence of replicated virus or plasmid vector was assayed by PCR using pWSRi.2236 and pWSRi.2556R primers. As expected, evidence of BCTV DNA was clearly detectable in pWSRi inoculated leaves after eight days, but was absent from pWSRiL1^- ^treated leaves (Figure 5). The absence of viral DNA in non-inoculated leaves from plants treated with pWSRi suggested that the modified viral genome remained confined to the inoculated leaf. In separate experiments, analysis of DNA extracts from tobacco protoplasts (BY2 cells) transfected with pWSRi and pWSRiL1^- ^confirmed that dsDNA corresponding to the modified BCTV genome (~2.5 kb) was released and replicated from pWSRi, and that the *L1 *mutation abolished replication (data not shown).

We asked whether non-replicating BCTV can sponsor silencing of an endogenous gene by constructing a pWSRiL1^-^:SpPI plasmids containing the identical *SpPI *insert previously shown to be effective when used in pWSRi:SpPI. The replication-defective pWSRiL1^-^:SpPI plasmid was inoculated to 12 plants. Five plants developed as normal females and seven plants developed as normal males. There were no transformed organs or modified flowers on any plants. We concluded that virus replication in inoculated cells is necessary to generate an effective systemic silencing signal, and that significant spread to non-inoculated cells is not required.

### Silencing of an endogenous nuclear gene in tomato

To further test the utility of the BCTV-based vector, and to determine if agroinfiltration might be used as an alternative delivery method, a binary Ti plasmid-based version of the vector (pWSRiA) was constructed and tested in tomato (*Solanum lycopersicum*). A targeting sequence directed against a 1 kb fragment of the sulfur allele (*su*) of magnesium chelatase from *Arabidopsis thaliana *was inserted to create pWSRiA:su. Comparison of homologous gene sequences from *Arabidopsis *and solanaceous species suggested that there was sufficient sequence identity to support at least partial silencing. As the *ChlI *allele is required for the synthesis of chlorophyll, loss of function results in leaf bleaching. A vector containing an irrelevant chloramphenicol acetyltransferase targeting sequence (pWSRiA:CAT) served as a negative control.

**Figure 6:**
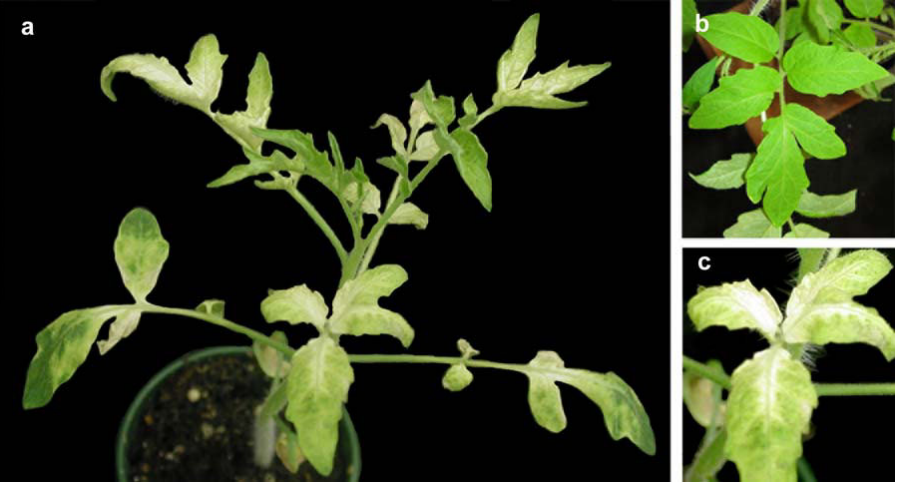
**Silencing phenotype obtained with pWSRiA:ChlI show a mosaic of leaf bleaching**. Tomato plants were agroinfiltrated with pWSRiA:ChlI to silence the sulfur allele of magnesium chelatase, or with pWSRiA:CAT (negative control). Photographs were taken approximately 4 weeks post-infiltration. (a) Plant treated with pWSRiA:ChlI. (b) Leaves of plant treated with pWSRiA:CAT. (c) Leaves of plant treated with pWSRiA:ChlI showing progressive bleaching beginning from the midrib.

In four independent experiments with a total of 15 inoculated plants, 12 of 15 plants agroinfiltrated at the two to four leaf stage with pWSRiA:ChlI exhibited bleaching indicative of silencing (Table 4). All 16 plants treated with pWSRiA:CAT were identical to untreated control plants. Bleaching was first observed in new leaves of pWSRiA:ChlI treated plants three to four weeks post-infiltration in the petioles and midribs, and spread distally through affected leaves. Bleaching was not uniform across the leaf surface, however, most leaves above the infiltration site were typically affected (Figure 6).

**Table 4:** Silencing of *Magnesium chelatase *(*su*): Bleaching response in treated tomato plants by trial.

Experiment	Plants silenced after treatment (silenced/infiltrated plants)	
	pWSRiA:CAT	pWSRiA:su
1	0/1	1/1
2	0/5	4/4
3	0/6	5/6
4	0/4	2/4

Tomato plants were agroinfiltrated with either pWSRiA:CAT (negative control) or with pWSRiA:ChlI to silence the sulfur allele of magnesium chelatase. Plants were scored for leaf bleaching indicative of silencing beginning 3 weeks post-infiltration.

To confirm that *ChlI *mRNA levels were altered by pWSRiA:ChlI, RNA extracts were obtained from three pWSRiA:ChlI treated plants, as well as from a plant treated with the control vector pWSRiA:CAT. The total cDNA was generated from the RNA samples using random hexamers. The relative amounts of *ChlI *mRNA were quantified by qRT-PCR using 18 S RNA as the internal controls (Table 5). The mean delta ct values were calculated as the mean of ct_*su*_- ct_18S _from the three replicates per RNA sample, and the variance of the delta ct values was estimated by summing the variances of the two individual ct values (*ChlI *and 18S) from the replicates. The mean delta ct values from the pWSRiA:ChlI plants were -0.3833 ± 0.1033, -1.0567 ± 0.0958, and -1.607 ± 0,1093, The delta ct from the pWSRiA:CAT was -2.6567 ± 0.2198. To obtain relative amounts of mRNA, we calculated the anti-log 2 of the delta ct and compared all values to the pWSRiA:CAT control value. The three pWSRiA:ChlI treated mRNA levels were 0.2068, 0.3299, and 0.4830 compared to the control. Thus, the *ChlI *mRNA was reduced in these leaves in the treated plants by approximately 50%–80%, and the phenotypes are associated with gene specific mRNA reductions.

**Table 5:** Silencing of *Magnesium chelatase *(*su*): Quantification of *Magnesium chelatase *(*su*) mRNA in three pWSRiA:ChlI versus control treatment

Treatment	Mean ct Mg Chelatase ± s.e.	Mean ct 18S ± s.e.	Delta ct ± 98% confidence limit	Relative Mg chelatase mRNA (2^Delta-Delta ct^)
pWSRiA:su	26.96 ± 0.05	27.34 ± 0.08	-0.38 ± 0.10	0.21

pWSRiA:su	24.67 ± 0.06	25.72 ± 0.06	-1.06 ± 0.10	0.33

pWSRiA:su	24.39 ± 0.08	25.64 ± 0.05	-1.61 ± 0.11	0.48

pWSRiA:CAT	25.26 ± .010	27.92 ± 0.17	-2.66 ± 0.22	1.00

Mean ct (threshold cycle) values were calculated from three replicates per cDNA sample. Delta ct values were calculated as the difference in ct values of *Mg chelatase *and *18S *amplifications for each sample. Relative *Mg chelatase *mRNA values were calculated as 2 to the power of the delta ct minus delta ct of the control (delta-delta values).

Although agroinfiltration is generally not an efficient method of gene delivery in tomato, these results allow us to conclude that pWSRi-based vectors can be effectively delivered by this method. This is likely due to the fact that the viral vector, once released from the plasmid vector, is highly amplified in inoculated cells.

### Penetrance and expressivity are target gene dependent

Throughout the trials, we noticed that the proportion of treated plants that demonstrated a gene-specific altered phenotype (comparable to penetrance) and the degree to which individual plants had altered phenotypes (comparable to expressivity) differed among the various target gene constructs. Silencing of floral organ identity transcription factors appeared to occur more frequently and with more consistent phenotypic effects than silencing of photosynthetic genes. We suspect that the level of gene expression and the longer half-life of the enzymatic proteins compared to the transcription factors may affect the level of penetrance and explain the somewhat less robust silencing observed in plants where *rbcS*, *transketolase*, or *ChlI *was targeted. Additionally, we have noted anecdotally that penetrance was lower at lower temperatures, as has been previously reported for other VIGS systems [8].

In all treatments, the silencing phenotypes varied spatially and temporally over the plant. For example, bleaching observed in pWSRiA:ChlI treated tomato plants appeared variegated, and in pWSRi:rbcS treated spinach plants, bleaching was often detected starting at the base of the leaf and spreading into the blade and along the midrib in late developing leaves (Figure 2C and Figure 6). In all cases, we observed the phenotypic changes first in organs that were not inoculated directly with the plasmid, and indeed had not developed at the time of application of the plasmid. Therefore the silencing signal must be transported to newly developing sink organs. We infer that the varied spatial and temporal appearance of the silencing effect reflects the time that the silencing signal (siRNA) takes to move systemically through the plant and the point of development of the affected meristem or organ when the signal arrives.

## Conclusion

In conclusion, we report the development of a silencing vector derived from the geminivirus BCTV and show that it is effective when used to target housekeeping genes or genes encoding transcription factors expressed in floral meristems. Replication, but not spread, of the virus vector is required for silencing, and we speculate that active BCTV replicons elicit strong and durable silencing signals that spread systemically and are efficiently amplified at distal sites by host silencing machinery. Regardless of mechanism, this separation of virus and target gene phenotypes, coupled with the extremely broad host range of BCTV and the flexibility in means of application, suggests that pWSRi-based vectors will prove to be useful probes of gene function in a wide variety of plant species.

## Competing interests

The authors declare that they have no competing interests.

## Authors' contributions

EMG and DMB conceived of the project and oversaw the research. EMG, DNS, and LCH carried out the construction and testing of the vector. EMG carried out the qRT-PCR analyses. KJB constructed the agro-infiltration modifications. KJB, NMV carried out testing in the tomato applications. All authors read and approved the final manuscript.

## References

[B1] Jorgensen RA, Cluster PD, English J, Que Q, Napoli CA (1996). Chalcone synthase cosuppression phenotypes in petunia flowers: comparison of sense vs. antisense constructs and single-copy vs. complex T-DNA sequences. Plant Molecular Biology.

[B2] Napoli CA, Lemieux C, Jorgensen RA (1990). Introduction of a chalcone synthase gene into Petunia results in reversible co-suppression of homologous genes in trans. Plant Cell.

[B3] Cogoni C, Macino G (2000). Post-transcriptional gene silencing across kingdoms. Current Opinion in Genetics and Development.

[B4] Hammond SM, Caudy AA, Hannon GJ (2001). Post-transcriptional gene silencing by double-stranded RNA. Nat Rev Genet.

[B5] Sharp PA (2001). RNA interference – 2001. Genes and Development.

[B6] Tabara H, Grishok A, Mello CC (1998). RNAi in C. elegans: soaking in the genome sequence. Science.

[B7] Waterhouse PM, Helliwell CA (2003). Exploring plant genomes by RNA-induced gene silencing. Nat Rev Genet.

[B8] Burch-Smith TM, Anderson JC, Martin GB, Dinesh-Kumar SP (2004). Applications and advantages of virus-induced gene silencing for gene function studies in plants. The Plant Journal.

[B9] Burch-Smith TM, Schiff M, Liu Y, Dinesh-Kumar SP (2006). Efficient Virus-Induced Gene Silencing in Arabidopsis. Plant Physiol.

[B10] Baulcombe D (1999). Viruses and gene silencing in plants. Arch Virol Suppl.

[B11] Carrillo-Tripp J, Shimada-Beltran H, Rivera-Bustamante R (2006). Use of geminiviral vectors for functional genomics. Curr Opin Plant Biol.

[B12] Huang C, Xie Y, Zhou X (2009). Efficient virus-induced gene silencing in plants using a modified geminivirus DNA1 component. Plant Biotechnol J.

[B13] Tao X, Zhou X (2004). A modified viral satellite DNA that suppresses gene expression in plants. Plant J.

[B14] Kjemtrup S, Sampson KS, Peele CG, Nguyen LV, Conkling MA, Thompson WF, Robertson D (1998). Gene silencing from plant DNA carried by a Geminivirus. Plant J.

[B15] Peele C, Jordan CV, Muangsan N, Turnage M, Egelkrout E, Eagle P, Hanley-Bowdoin L, Robertson D (2001). Silencing of a meristematic gene using geminivirus-derived vectors. Plant J.

[B16] Turnage MA, Muangsan N, Peele CG, Robertson D (2002). Geminivirus-based vectors for gene silencing in Arabidopsis. Plant J.

[B17] Hill JE, Strandberg JO, Hiebert E, Lazarowitz SG (1998). Asymmetric infectivity of pseudorecombinants of cabbage leaf curl virus and squash leaf curl virus: implications for bipartite geminivirus evolution and movement. Virology.

[B18] Stenger DC, Stevenson MC, Hormuzdi SG, Bisaro DM (1992). A number of subgenomic DNAs are produced following agroinoculation of plants with beet curly top virus. J Gen Virol.

[B19] Bennett CW (1971). The Curly Top Disease of Sugarbeet and Other Plants.

[B20] Thornberry HH (1966). Index of plant virus diseases: Plant pests of importance to North American agriculture.

[B21] Hormuzdi SG, Bisaro DM (1995). Genetic analysis of beet curly top virus: examination of the roles of L2 and L3 genes in viral pathogenesis. Virology.

[B22] Stenger DC (1994). Complete nucleotide sequence of the hypervirulent CFH strain of beet curly top virus. Mol Plant Microbe Interact.

[B23] Martin W, Mustafa AZ, Henze K, Schnarrenberger C (1996). Higher-plant chloroplast and cytosolic fructose-1,6-bisphosphatase isoenzymes: origins via duplication rather than prokaryote-eukaryote divergence. Plant Mol Biol.

[B24] Flechner A, Dressen U, Westhoff P, Henze K, Schnarrenberger C, Martin W (1996). Molecular characterization of transketolase (EC 2.2.1.1) active in the Calvin cycle of spinach chloroplasts. Plant Mol Biol.

[B25] Pfent C, Pobursky KJ, Sather DN, Golenberg EM (2005). Characterization of SpAPETALA3 and SpPISTILLATA, B Class Floral Identity Genes in Spinacia oleracea, and Their Relationship to Sexual Dimorphism. Development Genes and Evolution.

[B26] Vain P, Keen N, Murillo J, Rathus C, Nemes C, Finer JJ (1993). Development of the Particle Inflow Gun. Plant Cell, Tissue, and Organ Culture.

[B27] Stanley J, Latham JR, Pinner MS, Bedford I, Markham PG (1992). Mutational analysis of the monopartite geminivirus beet curly top virus. Virology.

[B28] Hormuzdi SG, Bisaro DM (1993). Genetic Analysis of Beet Curly Top Virus: Evidence for Three Virion Sense Genes Involved in Movement and Regulation of Single- and Double-Stranded DNA Levels. Virology.

[B29] Choi IR, Stenger DC (1996). The strain-specific cis-acting element of beet curly top geminivirus DNA replication maps to the directly repeated motif of the ori. Virology.

[B30] Hao L, Wang H, Sunter G, Bisaro DM (2003). Geminivirus AL2 and L2 proteins interact with and inactivate SNF1 kinase. Plant Cell.

[B31] Latham JR, Saunders K, Pinner MS, Stanley J (1997). Induction of plant cell division by beet curly top virus gene C4. The Plant Journal.

[B32] Sunter G, Sunter JL, Bisaro DM (2001). Plants Expressing Tomato Golden Mosaic Virus AL2 or Beet Curly Top Virus L2 Transgenes Show Enhanced Susceptibility to Infection by DNA and RNA Viruses. Virology.

[B33] Wang H, Buckley KJ, Yang X, Buchmann RC, Bisaro DM (2005). Adenosine kinase inhibition and suppression of RNA silencing by geminivirus AL2 and L2 proteins. J Virol.

[B34] Wang H, Hao L, Shung CY, Sunter G, Bisaro DM (2003). Adenosine kinase is inactivated by geminivirus AL2 and L2 proteins. Plant Cell.

[B35] Stenger DC, Revington GN, Stevenson MC, Bisaro DM (1991). Replicational release of geminivirus genomes from tandemly repeated copies: evidence for rolling-circle replication of a plant viral DNA. Proc Natl Acad Sci USA.

[B36] Frischmuth S, Frischmuth T, Latham JR, Stanley J (1993). Transcriptional analysis of the virion-sense genes of the geminivirus beet curly top virus. Virology.

[B37] Liu Y, Schiff M, Dinesh-Kumar SP (2002). Virus-induced gene silencing in tomato. Plant J.

[B38] Gossele V, Fache I, Meulewaeter F, Cornelissen M, Metzlaff M (2002). SVISS – a novel transient gene silencing system for gene function discovery and validation in tobacco plants. Plant J.

[B39] Theissen  G (2001). Development of floral organ identity: stories from the MADS house. Current Opinion in Plant Biology.

